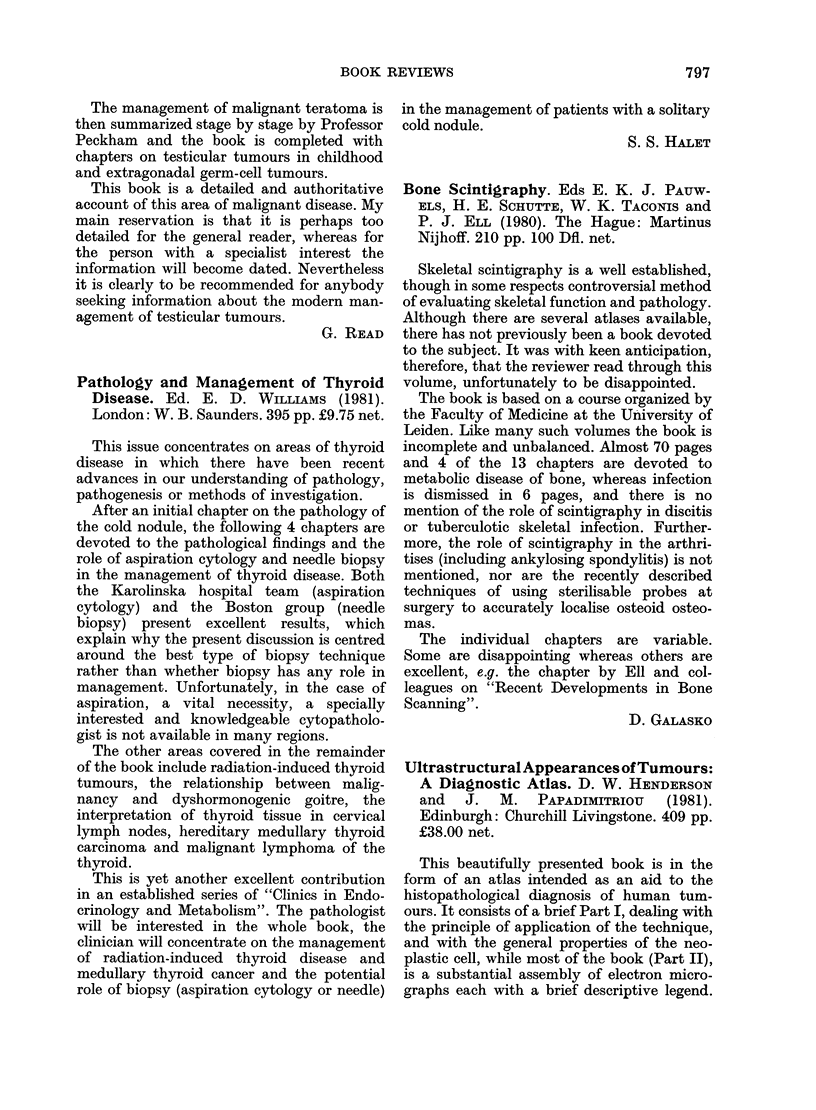# Bone Scintigraphy

**Published:** 1982-05

**Authors:** D. Galasko


**Bone Scintigraphy**. Eds E. K. J. PAUWELS, H. E. SCHUTTE, W. K. TACONIS and P. J. ELL (1980). The Hague: Martinus Nijhoff. 210 pp. 100 Dfl. net.

Skeletal scintigraphy is a well established, though in some respects controversial method of evaluating skeletal function and pathology. Although there are several atlases available, there has not previously been a book devoted to the subject. It was with keen anticipation, therefore, that the reviewer read through this volume, unfortunately to be disappointed.

The book is based on a course organized by the Faculty of Medicine at the University of Leiden. Like many such volumes the book is incomplete and unbalanced. Almost 70 pages and 4 of the 13 chapters are devoted to metabolic disease of bone, whereas infection is dismissed in 6 pages, and there is no mention of the role of scintigraphy in discitis or tuberculotic skeletal infection. Furthermore, the role of scintigraphy in the arthritises (including ankylosing spondylitis) is not mentioned, nor are the recently described techniques of using sterilisable probes at surgery to accurately localise osteoid osteomas.

The individual chapters are variable. Some are disappointing whereas others are excellent, *e.g.* the chapter by Ell and colleagues on "Recent Developments in Bone Scanning".

D. GALASKO